# Supplementary material for: Yiai Fuzheng decoction inhibits triple-negative breast cancer by remodeling the immune microenvironment
Source: Front Immunol. 2025 Sep 30;16:1615631. doi: 10.3389/fimmu.2025.1615631 (PMC12518410; doi:10.3389/fimmu.2025.1615631)
Supplement: Supplementary file 2 [file DataSheet1.zip › Supplementary File 2/Treatment/C_vs_D/07_Pathway_Analysis/Pathway_Analysis/Sig_Pathways_ID/Pyrimidine metabolism.pdf]

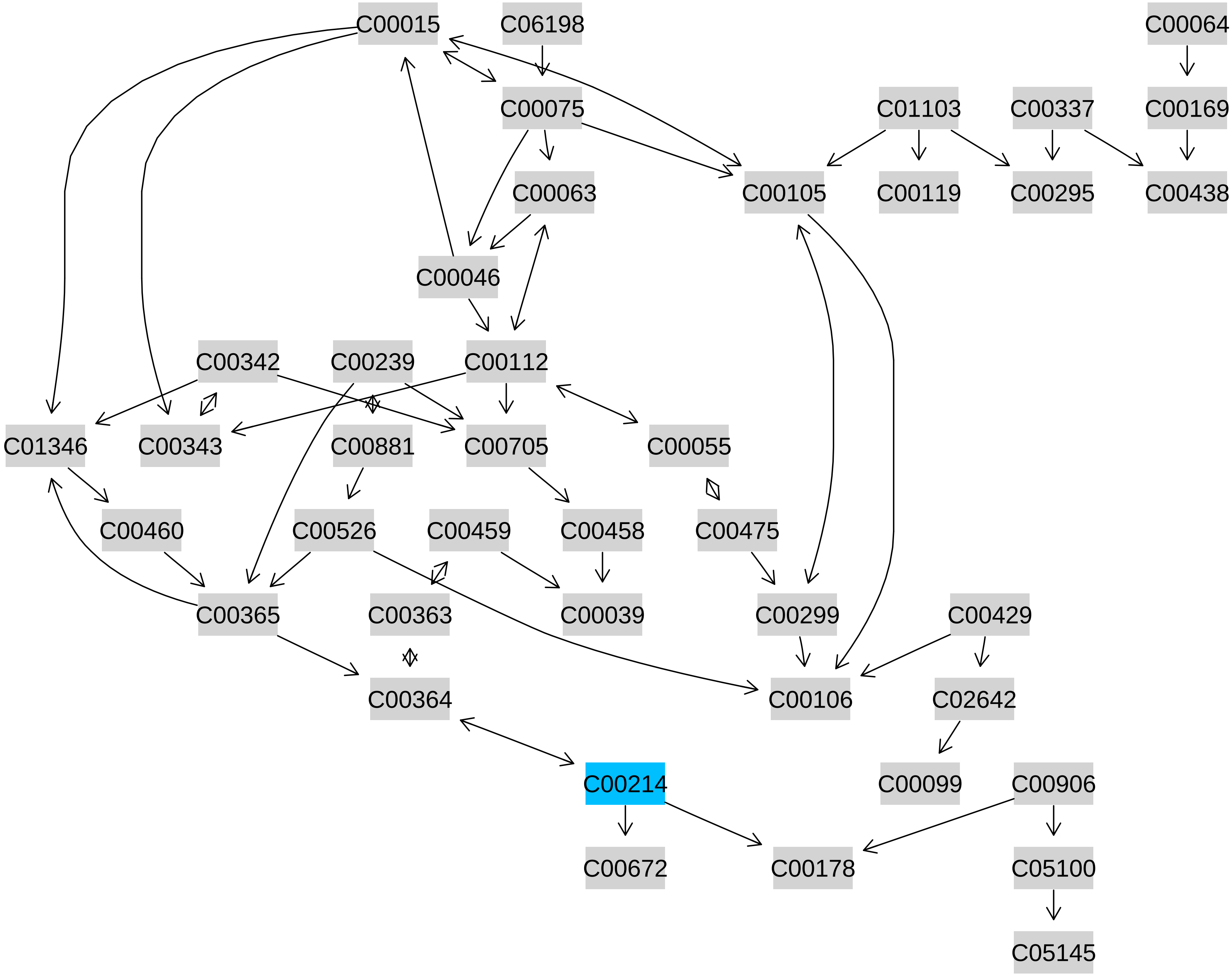
C00015
C06198
C00064
C00075
C01103
C00337
C00169
C00063
C00105
C00119
C00295
C00438
C00046
C00342
C00239
C00112
C01346
C00343
C00881
C00705
C00055
C00460
C00526
C00459
C00458
C00475
C00365
C00363
C00039
C00299
C00429
C00364
C00106
C02642
C00214
C00099
C00906
C00672
C00178
C05100
C05145
